# Whole-genome analysis reveals distinct adaptation signatures to diverse environments in Chinese domestic pigs

**DOI:** 10.1186/s40104-024-01053-0

**Published:** 2024-07-10

**Authors:** Zhen Wang, Bangmin Song, Jianyu Yao, Xingzheng Li, Yan Zhang, Zhonglin Tang, Guoqiang Yi

**Affiliations:** 1 Shenzhen Branch, Guangdong Laboratory of Lingnan Modern Agriculture, Key Laboratory of Livestock and Poultry Multi-Omics of MARA, Agricultural Genomics Institute at Shenzhen, Chinese Academy of Agricultural Sciences, Shenzhen, 518124 China; 2 Kunpeng Institute of Modern Agriculture at Foshan, Agricultural Genomics Institute at Shenzhen, Chinese Academy of Agricultural Sciences, Foshan, 528226 China; 3 School of Life Sciences, Henan University, Kaifeng, 475004 China; 4 Shenzhen Research Institute of Henan University, Shenzhen, 518000 China; 5 State Key Laboratory of Pharmaceutical Biotechnology, The University of Hong Kong, Hong Kong SAR, China; 6 Key Laboratory of Tropical Animal Breeding and Disease Research, Institute of Animal Science and Veterinary Medicine, Hainan Academy of Agricultural Sciences, Haikou, 571100 China; 7 Bama Yao Autonomous County Rural Revitalization Research Institute, Bama, 547500 China

**Keywords:** Environmental adaptation, Local Chinese breeds, Pig, Population genetics, Selection signals, Whole-genome resequencing

## Abstract

**Background:**

Long-term natural and artificial selection has resulted in many genetic footprints within the genomes of pig breeds across distinct agroecological zones. Nevertheless, the mechanisms by which these signatures contribute to phenotypic diversity and facilitate environmental adaptation remain unclear.

**Results:**

Here, we leveraged whole-genome sequencing data from 82 individuals from 6 domestic pig breeds originating in tropical, high-altitude, and frigid regions. Population genetic analysis suggested that habitat isolation significantly shaped the genetic diversity and contributed to population stratification in local Chinese pig breeds. Analysis of selection signals revealed regions under selection for adaptation in tropical (55.5 Mb), high-altitude (43.6 Mb), and frigid (17.72 Mb) regions. The potential functions of the selective sweep regions were linked to certain complex traits that might play critical roles in different geographic environments, including fat coverage in frigid environments and blood indicators in tropical and high-altitude environments. Candidate genes under selection were significantly enriched in biological pathways involved in environmental adaptation. These pathways included blood circulation, protein degradation, and inflammation for adaptation to tropical environments; heart and lung development, hypoxia response, and DNA damage repair for high-altitude adaptation; and thermogenesis, cold-induced vasodilation (CIVD), and the cell cycle for adaptation to frigid environments. By examining the chromatin state of the selection signatures, we identified the lung and ileum as two candidate functional tissues for environmental adaptation. Finally, we identified a mutation (chr1: G246,175,129A) in the *cis*-regulatory region of *ABCA1* as a plausible promising variant for adaptation to tropical environments.

**Conclusions:**

In this study, we conducted a genome-wide exploration of the genetic mechanisms underlying the adaptability of local Chinese pig breeds to tropical, high-altitude, and frigid environments. Our findings shed light on the prominent role of *cis*-regulatory elements in environmental adaptation in pigs and may serve as a valuable biological model of human plateau-related disorders and cardiovascular diseases.

**Supplementary Information:**

The online version contains supplementary material available at 10.1186/s40104-024-01053-0.

## Introduction

Environmental stressors can indeed have negative impacts on the health, welfare, and production efficiency of domesticated animals [1]. It is well established that environmental stress can significantly influence genome diversity [2]. As a result, identifying genomic features involved in the response to environmental pressure has become a focus of evolutionary biology. In natural populations, adaptation is a dynamic and long-term evolutionary process whereby populations enhance their adaptation by accumulating beneficial alleles at gene loci that control adaptive phenotypes [3]. Compared to natural populations, domesticated animals have experienced a greatly accelerated process of environmental adaptation via evolution due to human migration and selection [4]. Therefore, domestic animals represent an excellent model for probing adaptive mutations in current genetic studies.

Approximately 10,000 years ago, the domestication of pigs occurred in multiple regions across Eurasia [5, 6]. Since then, pigs have undergone significant domestication changes and have become one of the most economically domestic animals globally [7]. Compared to modern commercial pig breeds that are under strong selective pressure, such as Duroc and Landrace, indigenous pig breeds generally exhibit superior and broader environmental adaptability. Given the current and future impacts of climate change on the planet, understanding the genetic adaptability of local pig breeds to different environments and breeding highly adaptable pig breeds that can cope with climate change has practical value in modern pig farming and energy conservation. Furthermore, pigs share a high degree of physiological similarity with humans, particularly in terms of ecology, metabolism, and immunity [8]. Thus, exploring the biological processes related to environmental adaptation in domestic pigs could provide valuable insights into treating diseases caused by environmental factors in humans.

Recently, several genetic variations associated to environmental adaptation traits have been reported in multiple farm animals, including chickens [9], horses [10], sheep [11], cattle [12], bactrian camels [13, 14], and pigs [15–18]. These studies have provided valuable insights into the environmental adaptability and evolution of domestic animals, yet there has been limited research on the relationship between noncoding regions and adaptability. Given the ability of noncoding regions to regulate gene expression in a spatially and temporally specific manner, comprehensive studies integrating multi-tissue and multi-omics analyses are required to gain a deeper understanding of environmental adaptations.

In this study, whole-genome sequencing data were collected from 82 individuals from 6 Chinese native pig breeds according to our previous study. Based on these data, we conducted comprehensive analyses of the population structure and genome diversity of these pig breeds. Furthermore, we explored the potential relationships among swept genes, functional tissues, and candidate variants associated with adaptations to tropical, high-altitude, and frigid environments.

## Materials and methods

### Resequencing data collection

This study utilized a subset of resequencing data from project PRJNA754250 [19], comprising 82 individuals selected based on the environmental characteristics of their geographic origins. The study included Tibetan pigs from the Tibet plateau; Wuzhishan pigs, Ding'an pigs, and Tunchang pigs from Hainan Island; Hetao pigs from Inner Mongolia; and Min pigs from northeastern regions. Climate information associated to their habitats was retrieved from the study conducted by Zhao et al. [20]. We employed SOAPnuke v2.1.6 [21], a widely recognized tool for filtering raw reads, to remove adaptor and sequencing errors using the default parameters.

### Reads mapping of whole-genome sequencing data to the reference

The reads were first trimmed by fastp v0.23.0 [22] with default parameters. Next, all clean reads, including our newly generated samples, were aligned to the Sscrofa11.1 reference genome using the BWA-MEM pipeline [23]. The mapped reads were then sorted, and duplicates were removed by Picard tools v2.26.0 (https://broadinstitute.github.io/picard/) and SAMtools v1.14 [24].

### Genome-wide screening of SNPs and INDELs

The genome-wide variants were called for each sample by the GATK UnifiedGenotyper [25] with *-glm* BOTH *-rf* BadCigar *–sample_ploidy 2* option. To ensure high accuracy of variants calling, SNPs with *QD* < 2.0 || *FS* > 60.0 || *MQ* < 40.0 || *MQRankSum* < –12.5 || *ReadPosRankSum* < –8.0 were filtered. We then filtered out non-biallelic SNPs. After the quality screening, all the identified SNPs were further annotated using SnpEff v4.3t [26] based on the gene annotations of the pig reference genome Sscrofa11.1. Based on the genome annotation, SNPs were categorized as occurring in exonic regions, 5´ or 3´ untranslated regions, intronic regions, splicing sites (within 2 bp of a splicing junction), upstream and downstream regions (within a 1 kb region upstream or downstream from the transcription start site), or intergenic regions. SNPs in coding exons were further grouped as either synonymous SNPs or nonsynonymous SNPs. To check the confidence of SNPs called, we compared the SNPs identified with the dbSNP (https://www.ncbi.nlm.nih.gov/snp, last accessed Feb 23, 2021).

### Phylogenetic and population genetic analyses

To gain insights into phylogenetic relationships among pig breeds, we further filtered the SNP data set by applying criteria of minor allele frequency < 0.01, Hardy–Weinberg equilibrium < 0.001, and a proportion of missing genotypes > 10%. Subsequently, pruning was performed with the PLINK v1.90 [27] option "-indep-pairwise 50 5 0.2." Following filtering and linkage disequilibrium (LD) pruning, 1,358,458 SNPs were retained for subsequent population genetics analyses. A neighbor-joining (NJ) tree was constructed using the VCF2Dis v1.44 (https://github.com/BGI-shenzhen/VCF2Dis; accessed on 23 March 2022). The tree was displayed using the Interactive Tree Of Life (iTOL) [28]. To infer the population structure, we used ADMIXTURE v1.3.0 [29], which implements a block-relaxation algorithm. To identify the best K-value, the cross-validation error was tested for each K-value from 2 to 9. The principal component analysis (PCA) was conducted using the GCTA program [30]. The pattern of LD for these interest regions was computed using the PopLDdecay v3.40 [31].

### Selective sweeps during pig domestication and breeding

SNPs with minor allele frequency below 1% were removed from this analysis (Fig. S1). Considering that the vast majority of LD block sizes fall within the 0–50 kb range (98.48%) in our data (Fig. S2), a sliding-window approach (50-kb windows sliding in 10-kb steps) was applied to calculate the average SNP *F*_ST_ values (the Fixation Index, a measure in population genetics used to quantify genetic differentiation or genetic distance between populations) [32] and polymorphism levels (θπ, pairwise nucleotide variation as a measure of variability) [33, 34]. Windows containing ≥ 100 SNPs were used to detect signatures of selection sweeps. The *F*_ST_ was calculated using VCFtools v1.17 [35] with parameter "–weir-fst-pop group1 –weir-fst-pop group2 –fst-window-size 50,000 –fst-window-step 10,000". The θπ ratios were calculated using VCFtools v1.17 with parameters as follows: "–keep gropu1/gropu2 –window-pi 50,000 –window-pi-step 10,000". The XP-EHH (Cross Population Extended Haplotype Homozygosity) [36] was performed for every SNP using the default settings by selscan v2.0.0 [37], and genotypes were phased using Beagle 5.2 software [38] with default parameters. The test statistic was the average normalized XP-EHH score in each 50-kb region.

Those windows within the top 5% quantile of the two statistics were considered candidate selection targets and annotated using the genomic database search engine BioMart [39].

### Enrichment analysis

Previous studies have been mostly focused on traits of high value in animal production, like meat and carcass quality in pigs, leading to their overrepresentation in the QTL database. To counter this bias, we used a bootstrap simulation via the R package GALLO v1.4 [40] for QTL enrichment analyses against the pig QTL Database [41], accepting only *P*-values under 0.05 from multiple tests. Further, employing GALLO v1.4, we pinpointed genes in these regions against the Sscrofa11.1 reference genome assembly, extracting positional candidate genes for a more nuanced understanding of the genetic basis of diverse traits in pigs.

Gene Ontology (GO) enrichment analysis of swept genes was implemented with the R package clusterProfiler 4.0 [42]. We considered GO terms with corrected *P*-value < 0.05 to be significantly enriched.

We downloaded 15 chromatin states, including promoters (TssA, TssAHet, and TssBiv), TSS-proximal transcribed regions (TxFlnk, TxFlnkWk, and TxFlnkHet), enhancers (EnhA, EnhAMe, EnhAWk, EnhAHet, and EnhPois), repressed regions (Repr and ReprWk), quiescent regions (Qui), and accessible but did not coincide with any other measured epigenetic marks (ATAC islands) for 14 pig tissues (Adipose, Cecum, Cerebellum, Colon, Cortex, Duodenum, Hypothalamus, Ileum, Jejunum, Liver, Lung, Muscle, Spleen, and Stomach) from publicly available datasets [43]. We calculated the significance of enrichment based on Fisher's exact test using the R package LOLA v1.32.0 [44].

### Identification of putative functional SNPs

To identify putative functional SNPs, we first calculated *F*_ST_ by site and the top 1% sites located in specifically selected regions with high absolute allele frequency difference (ΔAF > 0.7) considered as candidate SNPs. The pCADD scores (a model based on the CADD methodology to create scores for the prioritisation of SNVs with respect to their putative deleteriousness in the genomes of wild and domesticated pigs) [45] were retrieved from public databases to prioritize coding variants. We downloaded the gene expression matrices of different pig breeds from publicly available datasets, PIGOME (http://pig123456789.pigome.com/). Motif analysis based on the JASPAR database [46] using the HOMER [47] for non-coding candidate SNPs located in the promoter or enhancer regions of candidate genes.

### Cell transfection and dual-luciferase reporter assays

A region of the *ABCA1* gene, encompassing 401 bp including the SNP (G-A, chr1: 246,175,129), was amplified and cloned into the pGL4.23-basic Luciferase Reporter Vector (GeneCreate, China). HEK293T cells (sourced from our laboratory's cell bank), resuspended in DMEM supplemented with 10% fetal bovine serum, were seeded into 12-well plates and cultured overnight at 37 °C in a 5% CO_2_ incubator. Upon reaching approximately 60% confluency, the cells were transfected with plasmids. The luciferase vector containing the SNP site (experimental vector; 1 μg) and the control reporter vector pRL-TK (20 ng) were co-transfected into the HEK293T cells at a 50:1 ratio using Attractene Transfection Reagent (Qiagen, Germany) according to the manufacturer's protocol. After 24 h of transfection, the transfected cells were collected and lysed. Luciferase activity was then measured using the Dual-Luciferase Reporter Assay System (Promega, USA), and the results were normalized to Renilla luciferase activity. Two-tailed Student's *t*-test was employed to determine the significance of differences, setting the significance threshold at *P* < 0.05.

## Results

### Genomic diversity, phylogenetic relationships and population structure

**Fig. 1:**
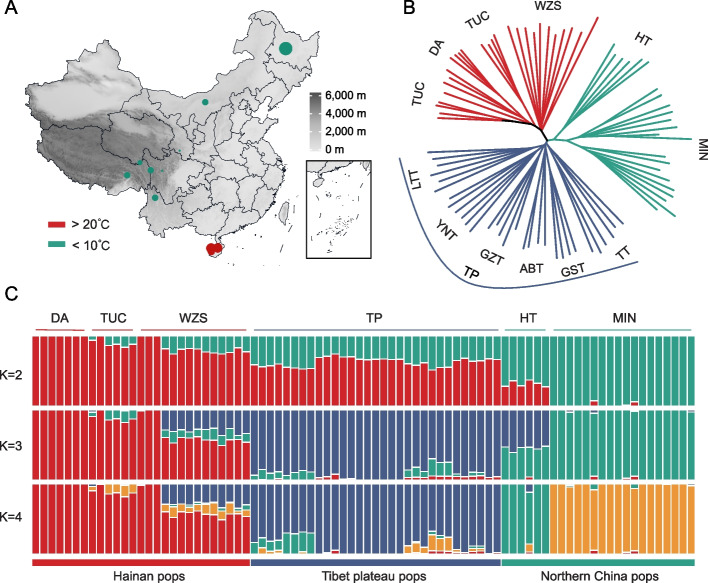
Geographic distribution and population genetics analyses of 6 domestic pig breeds. DA, Ding’an pigs; TUC, Tunchang pigs; WZS, Wuzhishan pigs; MZ, Min pigs; HT, Hetao pigs; TP, Tibetan pigs (YNT, TP in Yunnan; LTT, TP in Litang; GZT, TP in Ganzi; GST, TP in Gansu; ABT, TP in A’ba; TT, TP in Tibet). **A** Sampling locations of the 82 individuals in this study. The point size indicates the population size (4–18); the dot color indicates the annual average temperature. The elevation is shown on the map with a gradient. **B** The neighbor-joining tree was constructed based on whole-genome SNPs. **C** Population genetic structure of the 82 individuals. The number of assumed genetic clusters K ranged from 2 to 4 are shown

We collected data from 82 individuals of 6 Chinese native pig breeds, namely, Ding’an pigs (DA), Tunchang pigs (TUC), Wuzhishan pigs (WZS), Min pigs (MZ), Hetao pigs (HT), and Tibetan pigs (TP), which are spread across three classical geographical regions, i.e., tropical, high-altitude, and frigid environments (Fig. 1A and Table S1). All 82 individuals were sequenced at depths greater than 10 × . After applying stringent quality control criteria, we identified a total of 25,602,818 SNPs. By comparing the SNP set with the pig dbSNP database, we found that more than 13.5% of the variants (3,466,300 SNPs) were novel, which substantially expanded the catalog of porcine genetic variants (Fig. S3A). Further functional annotation revealed that most SNPs (64.12%) were located in intronic regions, followed by intergenic regions (22.82%). In addition, 1.21% of the SNPs were identified in coding regions, 102,024 of which were nonsynonymous variants (100,954 missense, 874 stop-gain, and 196 stop-loss mutants) (Fig. S3B).

LD generally decreased as the distance between loci increased, and the strength of LD varied widely between populations. The physical distance between SNPs, measured as half of the maximal value, was 34.1 kb (*r*^2^ = 0.34) for DA and 1.8–5.7 kb (*r*^2^ = 0.23–0.31) for the remaining five pig breeds. At longer marker distances, the LD value was highest for the DA but lowest for the Tibetan pigs (Fig. S4 and Table S3).

To infer the genetic and evolutionary relationships among pig breeds adapted to different environments, we first constructed a phylogenetic tree of 82 individuals using the NJ algorithm (Fig. 1B). The phylogenetic tree revealed distinct groupings of individuals from different regions. Specifically, the genetic relationships among the 6 breeds were strongly associated with their habitats, with three pig breeds from Hainan (DA, TUC, and WZS) exhibiting closer genetic relationships. Interestingly, within the Tibetan pig breed, the internal genetic relationships also showed significant geographical partitioning. The results of PCA were consistent with those of the phylogenetic analysis, with the first principal component (PC1 = 6.54%) and the second principal component (PC2 = 5.10%) able to separate the 6 breeds by geographical region (Fig. S5).

Population structure analysis revealed that the optimal number of clusters was three, at which point the cross-validation error was lowest, and the results were considered most reliable (Fig. 1C). When K = 2, the three pig breeds from Hainan (DA, TUC, and WZS), TP, and HT shared more ancestral components; when K = 3, we observed that pig breeds clustered by geographical region, consistent with the previous phylogenetic analysis and PCA results; when K = 4, HT were separated, consistent with the fact that their distribution area does not overlap with that of other pig breeds. The ADMIXTURE results further confirmed that the genetic relationships among pig breeds were closely related to their geographical distribution.

### Selection signatures on autosomes and functional annotation

To better leverage the diversity of our dataset, we partitioned the three local population samples into four groups based on the putative population structure: the high-temperature group (HP, 27 individuals consisting of DA, TUC, and WZS), the low-temperature group (NTP, 55 individuals consisting of HT, MZ, and TP), the high-altitude group (TP, 31 individuals consisting of Tibetan pigs), and the low-altitude group (HNP, 51 individuals consisting of DA, TUC, WZS, HT, and MZ). To elucidate the selective pattern of pigs in tropical environments, we conducted a comparative analysis between HP and NTP to detect selection signals. By applying the top 5% of the cutoffs for both *F*_ST_ and XP-EHH, we identified 55.50 Mb and 20.47 Mb selective sweep regions in HP (Table S4) and NTP, respectively. A similar approach was employed to compare TP and HNP to investigate their adaptive mechanisms in high-altitude environments, revealing 43.60 Mb selective sweep regions in TP (Table S5). To focus on the unique regions associated with adaptation to frigid environments, we excluded the overlapping regions between the NTP selection and TP selection from the NTP selection and ultimately identified 17.72 Mb regions (Table S6). The top 25 sweep regions with the highest *F*_ST_ and XP-EHH scores within the candidate genomic areas were considered highly relevant regions.

**Fig. 2:**
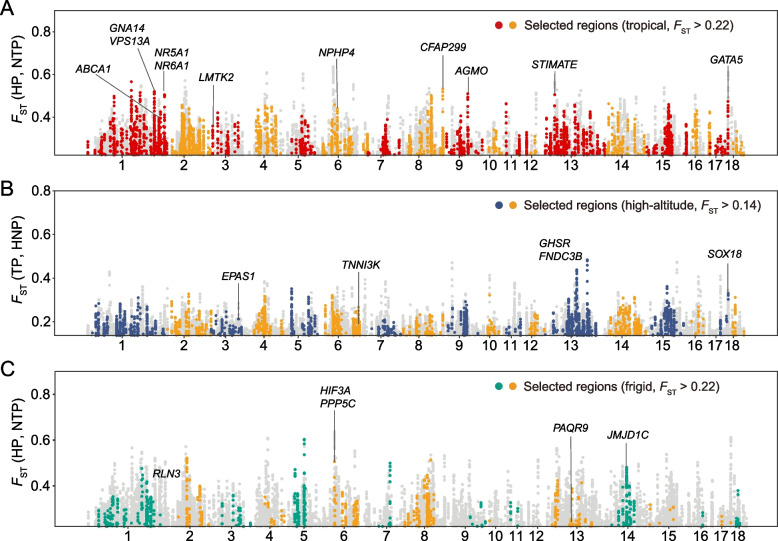
Genome-wide selective signals across environmental adaptations based on *F*_ST_ and XP-EHH. Candidate selection regions detected by two statistics (*F*_ST_ and XP-EHH) are plotted across the genome. All dots represent regions identified as outliers with the *F*_ST_ method. Red, blue, green, and yellow dots represent the regions identified as outliers in both methods. Genes in the selected regions were marked. **A** Selected regions of adaptation to tropical environments. **B** Selected regions of adaptation to high-altitude environments. **C** Selected regions of adaptation to frigid environments

We first performed an in-depth exploration of the selective patterns associated with adaptation to tropical environments (Fig. 2A). Most genes located in highly relevant regions are functional candidates for adaptation to tropical environments according to their annotations in previous studies. These genes included *VPS13A*, *GNA14*, and *NR6A1*, which are involved in blood coagulation and circulation [48]; *STIMATE* and *NR5A1*, which participate in the temperature stress response [49–51]; *AGMO*, which affects human inflammation and energy homeostasis [52]; *LMTK2,* which is associated with cell apoptosis [53]; and *CFAP299*, which may affect the hair phenotype of yaks [54]. Additionally, we noticed the *ABCA1* gene, located in the 54^th^ position among 2,254 candidate regions (Table S4), has been shown to reduce arteriosclerosis risk when up-regulated [55–57]. We explored the potential biological function of the detected signals with publicly available QTL and GO enrichment analyses. QTL enrichment analyses revealed that health, meat, and carcass traits were significantly enriched. We noticed significant enrichment for "Cholesterol level" and "Mean platelet volume" (Fig. S6A and Table S7). The positively selected genes (PSGs) involved in adaptation to tropical environments were mainly associated with blood circulation, protein degradation, and inflammation, including "blood vessel diameter maintenance" (*P* = 0.001046), "NIK/NF-kappaB signaling" (*P* = 0.010334), and "proteasomal ubiquitin-independent protein catabolic process" (*P* = 0.004777) (Fig. S6B and Table S8). Improving blood flow to the surrounding skin can mitigate the effects of heat stress in tropical environments [58]. Previous research has demonstrated that the NF-κB pathway can stimulate HSP activation in immune cells [59], contributing to reducing heat stress, and that the proteasomal ubiquitin-independent protein catabolic process can degrade misconfigured proteins caused by heat stress, reducing damage [60].

**Fig. 3:**
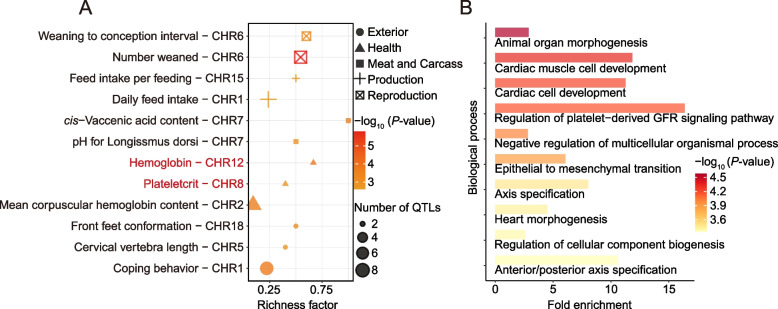
Annotation of the regions and genes under the high-altitude adaptation-specific selection based on the Animal QTLdb and the Gene Ontology Resource, respectively. **A** Significantly enriched QTL terms for high-altitude specific selection. The richness factor was obtained by calculating the ratio of the number of QTLs annotated in the candidate regions and the total number of each QTL. **B** Significantly enriched GO terms (Biological process, top 10) for selected genes in high-altitude specific selection

We next explored the mechanisms of hypoxia tolerance in Tibetan pigs (Fig. 2B). In the highly relevant regions of high-altitude adaptation-specific selection, two genes affecting the cardiovascular system were identified: *SOX18*, which is associated with the regulation of blood vessel development [61], and *TNNI3K*, which affects heart function  [62, 63]. Additionally, in line with previous research [64], the candidate gene *EPAS1* in the hypoxia-inducible factor pathway [65] showed substantial selection, but the gene *EGLN1* in the same pathway did not. By annotating specific selected regions, we discovered significant enrichment for blood index-associated traits, such as "Hemoglobin" and "Plateletcrit" (Fig. 3A and Table S9) Gene Ontology analysis revealed an overrepresentation of genes involved in biological processes that contribute to maintaining typical vital signs in high-altitude environments. (Fig. 3B and Table S10). PSGs detected in Tibetan pigs have particularly enriched in hypoxia adaptation-related processes, including "cardiac cell development" (*P* = 6.99E-05), "coronary vascular development" (*P* = 0.001084), "platelet-derived growth factor receptor signaling pathway" (*P* = 0.008778), "response to hypoxia" (*P* = 0.013765), and "respiratory tube development" (*P* = 0.000769). Additionally, we observed enrichment for the nucleotide metabolism process, including "pyrimidine-containing compound metabolic process" (*P* = 0.001629), "deoxyribose phosphate metabolic process" (*P* = 0.018889), and "GTP metabolic process" (*P* = 0.002461), which provided the basis for DNA repair and help to maintain genome stability by repairing UV-induced errors during DNA replication [66].

For adaptation to frigid environments, we detected four genes in the highly relevant regions, including *HIF3A*, which is associated with adiposity [67]; *JMJD1C*, which affects de novo lipogenesis [68]; *RLN3,* which is associated with food intake [69, 70]; and *PAQR9*, which activates brown adipocyte thermogenesis [71] (Fig. 2C). The function of these genes is critical for adaptation to frigid environments. The signal for adaptation to frigid environments was mainly associated with meat and carcass traits, especially "Fat area percentage in carcass", which may contribute to heat retention (Fig. S7A and Table S11). Biological process enrichment analysis revealed the process involved in thermogenesis "regulation of fibroblast growth factor receptor signaling pathway" (*P* = 0.004554). Elevated levels of FGF21 (fibroblast growth factor 21) promote beige adipose tissue and enhance energy expenditure [72, 73]. We also found that domestic pigs also exhibit cold-induced vasodilation (CIVD), as indicated by the enrichment of "vasodilation" (*P* = 0.008992). CIVD is a dramatic increase in peripheral blood flow observed during cold exposure. It supposedly protects against cold injuries [74]. Meanwhile, we noticed the enrichment for "cell cycle" (*P* = 0.002365), in line with the fact that the mammalian cell cycle is temperature sensitive [75] (Fig. S7B and Table S12).

### Annotation of variants candidate to be under selection for environmental adaptation

**Fig. 4:**
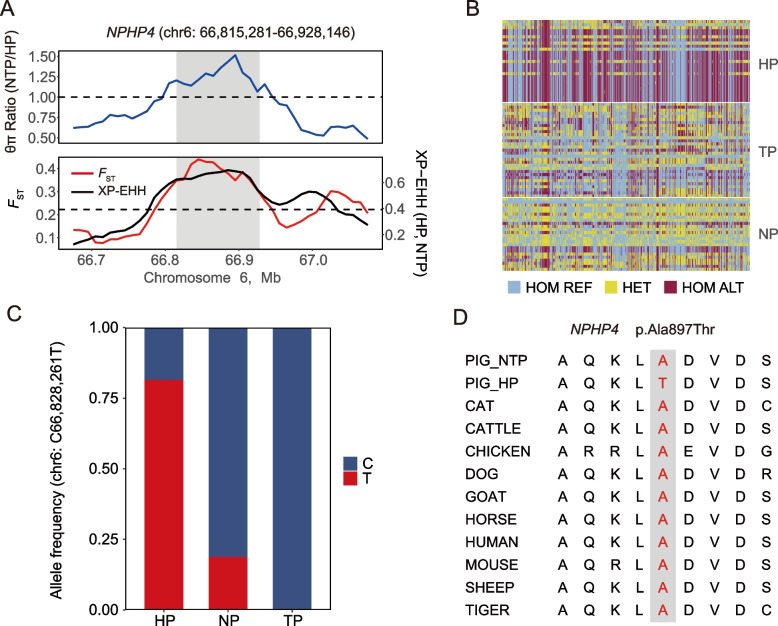
Candidate variant (p.Ala897Thr) in *NPHP4* for tropical adaptation. **A** θπ ratios (50-kb windows, 10-kb steps), *F*_ST_ values, and XP-EHH values around the *NPHP4* gene locus. The blue line represents θπ ratios. The red and black lines represent *F*_ST_ and XP-EHH values, respectively. **B** Haplotype pattern in the genomic region of *NPHP4* among HP, TP, and NP. **C** Allele frequency of the mutation site. **D** Multispecies alignment of the protein sequences around the variant. HP, consisting of Ding’an pigs, Tunchang pigs, and Wuzhishan pigs; NP, consisting of Hetao pigs and Min pigs; TP, Tibetan pigs

To better interpret the genetic basis of domestic selection, we annotated the SNPs within selected regions. We identified 10 nonsynonymous variants with high pCADD values (pCADD > 10) in regions specifically selected for adaptation to tropical environments (Table S13). For example, we identified the mutation p.Val244Gly in *VPS13A*, which was previously reported as a mutation that may impact the secretion and aggregation of blood platelets and reduce the risk of thrombosis in southern Chinese pigs from hot environments [15] (Fig. S8A). Additionally, in *NPHP4*, which has been verified by *F*_ST_, XP-EHH, θπ ratio, and genotype patterns as a positively selected gene for adaptation to tropical environments (Fig. 4A–C), we found a nonsynonymous variant (p.Ala897Thr) that showed a large difference in allele frequency between HP and NTP; this was predicted to be a functional-altering variant and was found to be highly conserved across multiple vertebrate species (Fig. 4D). *NPHP4* is a cilia-associated protein that negatively regulates the mammalian Hippo signaling pathway and is linked to severe degenerative renal disease, nephronophthisis and blindness in humans [76, 77]. We propose that the missense mutation in *NPHP4* may enhance water reabsorption in the kidney to mitigate the effects of heat stress on pigs.

To evaluate whether these putative promising variants identified in this study are originated from novel mutations or standing variants from the early stage of domestication, we referenced resequencing data from project PRJNA754250, which includes 33 Asian wild boars (AW) and 33 European wild boars (EW), covering diverse subpopulations (Table S2). Our allele frequency analysis identified a missense mutation (p.Asp2,905Asn, pCADD = 19.41) in *VPS13B* prevalent in tropical populations, which was also found in wild populations (Fig. S8B–C and Table S13). This suggested it may be a standing variant that became dominant as pigs were domesticated in hot areas, potentially playing a role in tropical adaptation, given the *VPS13B* is closely related to *VPS13A* within the same gene family.

### Chromatin state analysis enhanced the biological interpretations of adaptive evolution

**Fig. 5:**
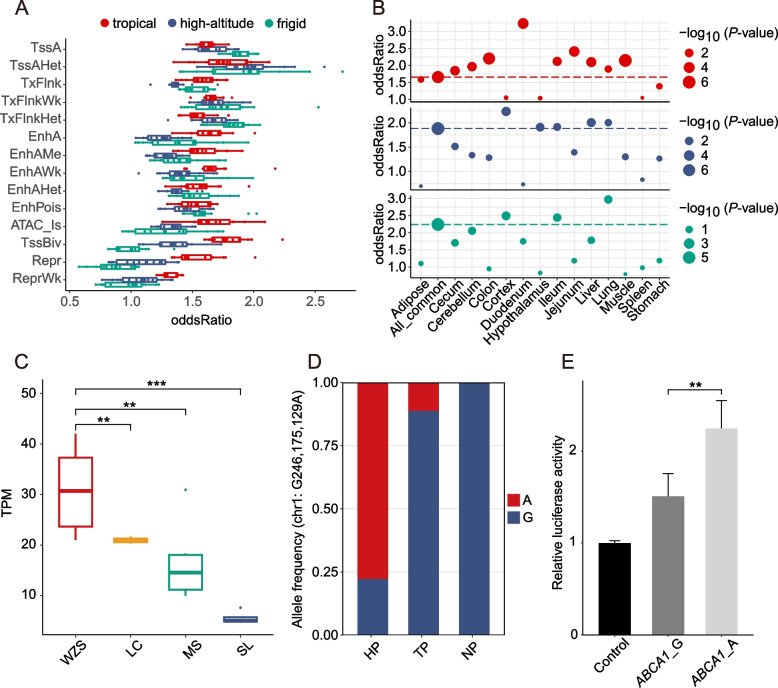
Chromatin state plays an important role in the environmental adaptations of Chinese domestic pigs. **A** Enrichment of 14 chromatin states in candidate genomic regions of adaptation to tropical, high-altitude, and frigid environments. Each point represents a tissue, and oddsRatio displays the enrichment intensity of each chromatin state. **B** Enrichment of tissue-specific promoters (TssA) candidate genomic regions of adaptation to tropical, high-altitude, and frigid environments. Using the enrichment intensity of All_common promoter as a reference to divide the enrichment intensity of each tissue-specific promoter. **C** Expression of *ABCA1* gene in liver of different pig breeds. From left to right, the average annual temperature of pig breeding areas is decreasing. WZS, Wuzhishan pigs; LC, Luchuan pigs; MS, Meishan pigs; SL, Songliao pigs. Climate information can be found in Table S1. The significance of the genotype difference is tested. ^**^P < 0.05 (*t*-test), ^***^P < 0.001 (*t*-test). **D** Allele frequency of the mutation site in the intron of *ABCA1* (chr1: G246,175,129A). HP, consisting of Ding’an pigs, Tunchang pigs, and Wuzhishan pigs; NP, consisting of Hetao pigs and Min pigs; TP, Tibetan pigs. **E** Luciferase reporter assays in HEK-293T cells to compare enhancer activity between the two alleles. ^**^P < 0.05 (*t*-test)

Tissue-specific gene regulation plays a crucial role in the process of adaptive evolution [43]. Thus, we performed chromosome state enrichment analysis for the genomic regions under selective pressure within tropical, high-altitude, and frigid environments (Fig. 5A and Tables S14–S16). The results showed a high consistency: TssA and TSS-proximal transcribed regions were most enriched, followed by enhancers. Then we examined the tissue-specific promoters (TssA) (Fig. 5B and Tables S17–S19). Using a common promoter as a reference, our analysis revealed that lung-specific and ileum-specific promoters were preferentially enriched in all the three types of adaptation. Interestingly, most tissue-specific promoters in this study were preferentially enriched in adaptation to tropical environments. This indicated that heat stress affects a wide range of tissues. Several tissue-specific promoters were found to be preferentially enriched in one or more types of selection, including liver-specific promoters involved in tropical/high-altitude adaptation and cortex-specific promoters involved in frigid/high-altitude adaptation. Additionally, spleen-specific and stomach-specific promoters were not preferentially enriched in any of the three types of adaptation.

### Variation within the *cis*-regulatory regions involving tropical adaptation

The *ABCA1* gene was positively correlated in adaptation to tropical environments according to the *F*_ST_, XP-EHH, θπ ratio, and genotype patterns (Fig. S9A**–**B). To investigate whether the expression of the *ABCA1* gene was associated with the environmental origins of pig breeds, we analyzed gene expression data (Table S20). RNA-seq data showed that *ABCA1* expression was elevated in pig liver (Fig. S9C) and positively correlated with the annual mean temperature of the breed's origin (Fig. 5C). This trend was specific to liver tissue and was not observed in other tissues (Fig. S9D–E). Based on the chromatin state data, we found a variant (chr1: 246,175,129, G-to-A) in the intron of *ABCA1* that may regulate expression, as it is located in the enhancer region. The SNP showed a higher allele frequency in HP (77.8%) than in TP (11.3%) and NP (0). HOMER analysis revealed that the G-to-A mutation might alter the transcription factor binding motif at this position (Fig. S10 and Table S21). Subsequent luciferase reporter assays revealed higher enhancer activity of the *ABCA1* gene segment that including the A allele (Fig. 5E). Therefore, we inferred that the mutation enhanced the expression of *ABCA1*, which may contribute to adaptation to tropical environments.

## Discussion

Domestic pigs are important agricultural animals, serving as a substantial source of animal protein globally. Intense artificial selection and crossbreeding have increased the productivity of modern commercial pig breeds but reduced their adaptive potential [78, 79]. With the changing global climate, studying the genetic adaptations of local breeds to diverse environments is crucial. In this study, we conducted a comprehensive investigation of the environmental adaptability of Chinese domestic pigs using whole-genome sequencing data and multiple omics datasets. Our findings underscore the importance of understanding the adaptive potential of domestic pigs to environmental challenges and have significant implications for the breeding of highly adaptable pig breeds.

### Population genetic analysis

Genomic analyses revealed differences among pig breeds from distinct geographic regions, with local Chinese breeds likely originating from ancient Yellow River basin domestication centers [80–83]. Compared to modern breeds, Chinese local pigs, particularly Tibetan pigs, exhibit faster linkage disequilibrium decay, though slower than wild boars [84–87]. This suggested that after domestication, local pig populations spread with human migration to diverse agricultural zones and were shaped by combined artificial and natural selection or gene flow. The genomic diversity of local Chinese pig breeds was closely associated with breeding practices, such as the free-range breeding of Tibetan pigs by Tibetans, which may have increased gene flow with local wild boars and resulted in faster linkage disequilibrium decay.

### Functional annotation

Annotation against a single database alone cannot fully reveal the primary roles of genes within regions under selection in an organism. To explore the functions of candidate genes, we employed a multifaceted approach combining GO enrichment and QTL enrichment analyses. Our analysis confirmed a significant enrichment of traits related to blood circulation in both tropical and high-altitude adaptation in pigs, consistent with prior investigations of blood biochemical indicators in heat-stressed pigs and Tibetan pigs under normal conditions [88–90]. However, upon further analysis of the specific enriched QTLs ("LDL cholesterol" and "Cholesterol level" for adaptation to tropical environments; "Red blood cell count", "Red cell distribution width", and "Hemoglobin" for high-altitude adaptation) and specific enriched GO terms, we found that the overlap was coincidental. Pigs adapted to the tropics need to increase blood circulation for heat dissipation and to reduce the risk of thrombosis [91, 92]. In contrast, pigs adapted to high altitudes need to reduce blood viscosity caused by high hemoglobin levels and thus reduce cardiac burden [93, 94]. This parallel selection of traits warrants consideration in the breeding of pigs with broad environmental adaptability. In contrast to the parallel selection of traits, specific selection for adaptation to distinct environments is more prevalent. Our results support this notion, as evidenced by the selection for fat coverage in pig breeds from cold regions, granulocyte activity and blood lipid content in tropical pig breeds, and hemoglobin content in pig breeds from high-altitude hypoxic environments. These specifically selected traits may facilitate the breeding of pig breeds adapted to particular environments.

### Chromatin state annotation and *cis*-regulatory mutations

Gene regulation plays a crucial role in speciation and adaptive diversification [95–97]. *cis*-Regulatory mutations can alter the expression of proximal genes and have long been considered important targets for adaptive phenotypic evolution, as they may have fewer deleterious effects than changes in protein-coding sequences [98–100]. While protein-coding mutations may affect protein products throughout tissues and developmental stages, *cis*-regulatory mutations can influence gene expression in a spatially and temporally specific manner. Several studies have identified the importance of noncoding region mutations in local adaptation [101]. Previous research on the adaptive evolution of domestic pigs has focused primarily on protein-coding regions of the genome, annotating candidate gene functions to elucidate environmental adaptation, with little systematic exploration of regulatory regions.

To investigate the mechanisms by which variation in regulatory regions affects the environmental adaptability of pigs, we analyzed tissue-specific chromatin states in candidate regions. By examining the enrichment of tissue-specific regulatory factors, we pinpointed the lung and ileum as common functional tissues for adaptation to tropical, high-altitude, and frigid environments. The role of the lung, the undertaker of respiration, in the process of adapting to various environments has been well studied [102–104]. The ileum is the exclusive site for vitamin B_12_ absorption, crucial for maintaining energy production and stress response under environmental changes [105]. As the main intestinal segment where Peyer’s patches are distributed, ileum plays a significant role in immune homeostasis by regulating gut microbiota through interactions involving IgA and epithelial barriers, enhancing disease resistance and environmental adaptability [106–109]. Additionally, the interactions between the host and the gut microbiome influences host metabolic processes and adaptability to diverse climates [110–116], underscoring the pivotal role of ileum in physiological resilience. In contrast, the spleen and stomach did not appear to have a specific role in the adaptation of domestic pigs to tropical, high-altitude, or frigid environments.

Our investigation demonstrated a possible association between the *ABCA1* gene and tropical adaptation in domestic pigs. The identified SNP (chr1: 246,175,129, G-to-A), situated within the *cis*-regulatory region of *ABCA1*, exhibits genotype frequencies correlated with warmer climates. However, the precise mechanisms through which this SNP influences *ABCA1* expression—and thereby contributes to tropical adaptation—remain unclear. To substantiate this hypothesis, further studies investigating the three-dimensional interactions between the SNP and the *ABCA1* gene, alongside comprehensive animal experiments, are essential for a more definitive understanding of the gene’s role in environmental adaptability.

Through the elucidation of the positive selection phenomena occurring within regulatory regions during environmental adaptation, our study extended our understanding of pig environmental adaptability to specific tissues, providing a framework for incorporating single-cell data into future adaptability research while also highlighting the crucial role played by *cis*-regulatory mutations in enabling pig adaptation to tropical environments.

## Conclusion

This genome-wide study revealed the genetic mechanisms behind the adaptation of Chinese pig breeds to tropical, high-altitude, and frigid environments. Our findings revealed significant population stratification driven by habitat isolation and pinpointed genomic regions under selection linked to key adaptive traits. Notably, we identified candidate functional tissues for adaptation include the lung and ileum and uncovered a mutation (chr1: G246,175,129A) in the *cis*-regulatory region of *ABCA1* gene as a promising variant for tropical adaptation. Overall, the genetic diversity observed, encompassing both coding and non-coding regions, underscored the complex genetic basis of environmental tolerance and survival. Our results provided profound insights into the genetic architecture of environmental adaptation, offering valuable implications for pig breeding programs and serving as a reference for understanding human diseases related to environmental stress.

### Supplementary Information

**Additional file 1**: **Fig. S1**. Genome-wide distribution of SNPs. Out of 226,375 windows of 50 kb in length sliding in 10 kb steps across the domestic pig genome, 5,413 windows contain < 100 SNPs (red bars) and cover 2.39% of the genome (dashed lines). 220,096 windows contain ≥ 100 SNPs (blue bars) and cover 97.61% of the genome, and these were used to detect signatures of selective sweeps. The cumulative % in whole genome length (black line) is also charted. **Fig. S2**. The block size distribution for each autosome in our data. **Fig. S3**. Genetic variants of 82 genomes from 6 local Chinese pig breeds (Ding’an pigs; Hetao pigs; Min pigs; Tibetan pigs; Tunchang pigs; Wuzhishan pigs). **A** Venn diagrams for novel variants detected in domestic pigs. **B** Annotation of 25,602,818 SNPs retrieved from domestic pigs. **Fig. S4**. Decay of Linkage disequilibrium (LD) for 6 breeds, with one line per breed. (DA, Ding’an pigs; HT, Hetao pigs; MZ, Min pigs; TP, Tibetan pigs; TUC, Tunchang pigs; WZS, Wuzhishan pigs). **Fig. S5**. A principal component plot of the 82 individuals based on SNP information. The color represents the location of the pig breeds. DA, Ding’an pigs; HT, Hetao pigs; MZ, Min pigs; TP, Tibetan pigs; TUC, Tunchang pigs; WZS, Wuzhishan pigs. **Fig. S6**. Annotation of the regions and genes under tropical adaptation-specific selection based on the Animal QTLdb and the Gene Ontology Resource, respectively. **A** Significantly enriched QTL terms for tropical-specific selection. **B** Significantly enriched GO terms (Biological process, top 10) for tropical-specific selection. **Fig. S7**. Annotation of the regions and genes under frigid adaptation-specific selection based on the pig QTLdb and the Gene Ontology Resource, respectively. **A** Significantly enriched QTL terms for frigid-specific selection. **B** Significantly enriched GO terms (Biological process, top 10) for frigid-specific selection. **Fig. S8**. Multispecies regional alignment of the *VPS13A* and *VPS13B* protein sequences around candidate variants for adaptation to tropical environments. **A** p.Val244Gly in *VPS13A* gene. **B** p.Asp2,905Asn in *VPS13B* gene. **C** Allele frequency of the putative promising variant in VPS13B among different breeds. AW, Asian wild pigs; EW, European wild boars; AWNC, Northern Chinese wild boars; AWSC, Southern Chinese wild boars. **Fig. S9**. Distinct genomic landscape around *ABCA1* gene. **A** θπ ratios (50-kb windows, 10-kb steps), *F*_ST_ values, and XP-EHH values around the *ABCA1* gene locus. The blue line represents θπ ratios. The red and black lines represent *F*_ST_ and XP-EHH values, respectively. **B** Haplotype pattern in the genomic region of *ABCA1* among HP, TP, and NP. HP, consisting of Ding’an pigs, Tunchang pigs, and Wuzhishan pigs; TP, Tibetan pigs; NP, consisting of Hetao pigs and Min pigs. **C** The *ABCA1* gene shows high expression in pig liver. **D** and **E** Expression of *ABCA1* gene in adipose and muscle tissues of different pig breeds. From left to right, the average annual temperature of pig breeding areas is decreasing. The significance of the genotype difference is tested. ^**^P < 0.05 (*t*-test), ^***^P < 0.001 (*t*-test). **Fig. S10**. Sequence logos of *NFIA* and *MEIS2*. Site 7 (G allele) of *NFIA* and site 4 (A allele) of *MEIS2* are the binding sites of chr1: 246,175,129.

**Additional file 2: Table S1**. Samples, origin, and environmental variables of Chinese pigs in this study. **Table S2**. The sequencing quality and read alignment statistic of whole-genome sequencing data in this study. **Table S3**. The average value of LD in different distance regions among 6 domestic pig breeds. **Table S4**. List of regions under selection for adaptation to tropical environments and enumeration of the genes within such regions. **Table S5**. List of regions under selection for adaptation to high-altitude environments and enumeration of the genes within such regions. **Table S6**. List of regions under selection for adaptation to frigid environments and enumeration of the genes within such regions. **Table S7**. Fold enrichment of tropical adaptation-specific selection signatures for pig QTL terms. **Table S8**. Significantly enriched GO terms of the genes in tropical adaptation-specific selection regions. **Table S9**. Fold enrichment of high-altitude adaptation-specific selection signatures for pig QTL terms. **Table S10**. Significantly enriched GO terms of the genes in high-altitude adaptation-specific selection regions. **Table S11**. Fold enrichment of frigid adaptation-specific selection signatures for pig QTL terms. **Table S12**. Significantly enriched GO terms of the genes in frigid adaptation-specific selection regions. **Table S13**. Candidate nonsynonymous variants (ΔAF > 0.7) of tropical adaptation-specific selection with high pCADD score. **Table S14**. Fold enrichment of tropical adaptation-specific selection signatures for chromatin states. **Table S15**. Fold enrichment of high-altitude adaptation-specific selection signatures for chromatin states. **Table S16**. Fold enrichment of frigid adaptation-specific selection signatures for chromatin states. **Table S17**. Fold enrichment of tropical adaptation-specific selection signatures for tissue-specific promoters. **Table S18**. Fold enrichment of high-altitude adaptation-specific selection signatures for tissue-specific promoters. **Table S19**. Fold enrichment of frigid adaptation-specific selection signatures for tissue-specific promoters. **Table S20**. *ABCA1* expression levels across various tissues in different pig breeds (Download from http://pig123456789.pigome.com/). **Table S21**. Prediction of the transcription factors binding around the SNP (chr1:246,175,129).

## Data Availability

The whole genome resequencing data that were analyzed during the current study are available in the NCBI primary data archive (PDA) with accession number PRJNA754250. Chromatin state data used in this study can be found in: http://farm.cse.ucdavis.edu/~zhypan/Nature_Communications_2021. The RNA-seq data is available in the publicly available datasets, PIGOME (http://pig123456789.pigome.com/).

## Abbreviations

AF: Allele frequency
CIVD: Cold-induced vasodilation
EnhA: Strong active enhancer
EnhAHet: Active enhancer no ATAC
EnhAMe: Medium enhancer with ATAC
EnhAWk: Weak active enhancer
EnhPois: Poised enhancer
GO: Gene Ontology
LD: Linkage disequilibrium
MAF: Minor allele frequency
NJ: Neighbor-Joining
PCA: Principal component analysis
pCADD: Combined annotation dependent depletion for pig
PCR: Polymerase chain reaction
PSG: Positively selected gene
QTL: Quantitative trait locus
Qui: Quiescent regions
Repr: Repressed polycomb
ReprWk: Weak repressed polycomb
SNP: Single nucleotide polymorphism
TssA: Strongly active promoters/transcripts
TssAhet: Flanking active TSS without ATAC
TssBiv: Bivalent/Poised TSS
TxFlnk: Transcribed at gene
TxFlnkHet: Transcribed region without ATAC
TxFlnkWk: Weak transcribed at gene
