# Supplementary material for: Whole-genome analysis reveals distinct adaptation signatures to diverse environments in Chinese domestic pigs
Source: J Anim Sci Biotechnol. 2024 Jul 10;15:97. doi: 10.1186/s40104-024-01053-0 (PMC11234542; doi:10.1186/s40104-024-01053-0)
Supplement: Supplementary file 1 — Additional file 1: Fig. S1. Genome-wide distribution of SNPs. Out of 226,375 windows of 50 kb in length sliding in 10 kb steps across the domestic pig genome, 5,413 windows contain < 100 SNPs (red bars) and cover 2.39% of the genome (dashed lines). 220,096 windows contain ≥ 100 SNPs (blue bars) and cover 97.61% of the genome, and these were used to detect signatures of selective sweeps. The cumulative % in whole genome length (black line) is also charted. Fig. S2. The block size distribution for each autosome in our data. Fig. S3. Genetic variants of 82 genomes from 6 local Chinese pig breeds (Ding’an pigs; Hetao pigs; Min pigs; Tibetan pigs; Tunchang pigs; Wuzhishan pigs). A Venn diagrams for novel variants detected in domestic pigs. B Annotation of 25,602,818 SNPs retrieved from domestic pigs. Fig. S4. Decay of Linkage disequilibrium (LD) for 6 breeds, with one line per breed. (DA, Ding’an pigs; HT, Hetao pigs; MZ, Min pigs; TP, Tibetan pigs; TUC, Tunchang pigs; WZS, Wuzhishan pigs). Fig. S5. A principal component plot of the 82 individuals based on SNP information. The color represents the location of the pig breeds. DA, Ding’an pigs; HT, Hetao pigs; MZ, Min pigs; TP, Tibetan pigs; TUC, Tunchang pigs; WZS, Wuzhishan pigs. Fig. S6. Annotation of the regions and genes under tropical adaptation-specific selection based on the Animal QTLdb and the Gene Ontology Resource, respectively. A Significantly enriched QTL terms for tropical-specific selection. B Significantly enriched GO terms (Biological process, top 10) for tropical-specific selection. Fig. S7. Annotation of the regions and genes under frigid adaptation-specific selection based on the pig QTLdb and the Gene Ontology Resource, respectively. A Significantly enriched QTL terms for frigid-specific selection. B Significantly enriched GO terms (Biological process, top 10) for frigid-specific selection. Fig. S8. Multispecies regional alignment of the VPS13A and VPS13B protein sequences around candidate variants fo [file 40104_2024_1053_MOESM1_ESM.docx]

**
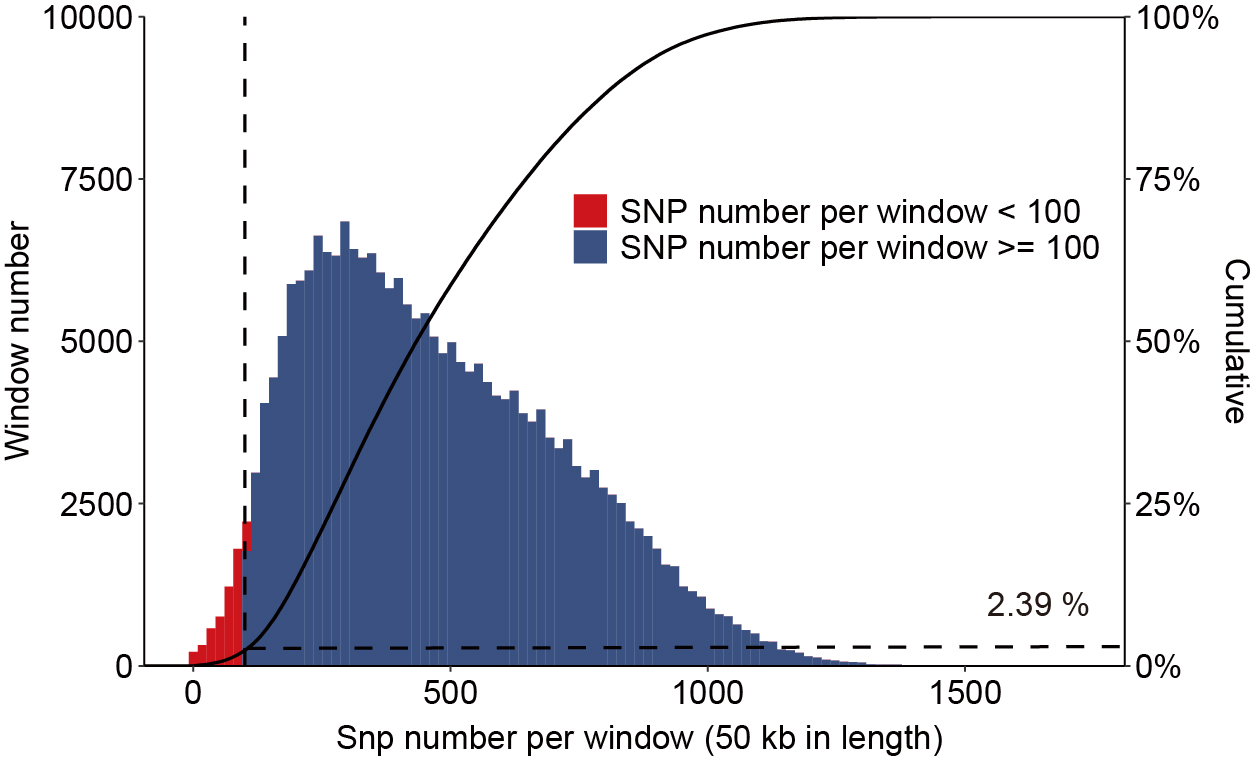
**

**Fig. S1.** Genome-wide distribution of SNPs. Out of 226,375 windows of 50 kb in length sliding in 10-kb steps across the domestic pig genome, 5,413 windows contain < 100 SNPs (red bars) and cover 2.39% of the genome (dashed lines). 220,096 windows contain ≥ 100 SNPs (blue bars) and cover 97.61% of the genome, and these were used to detect signatures of selective sweeps. The cumulative % in whole genome length (black line) is also charted.

**
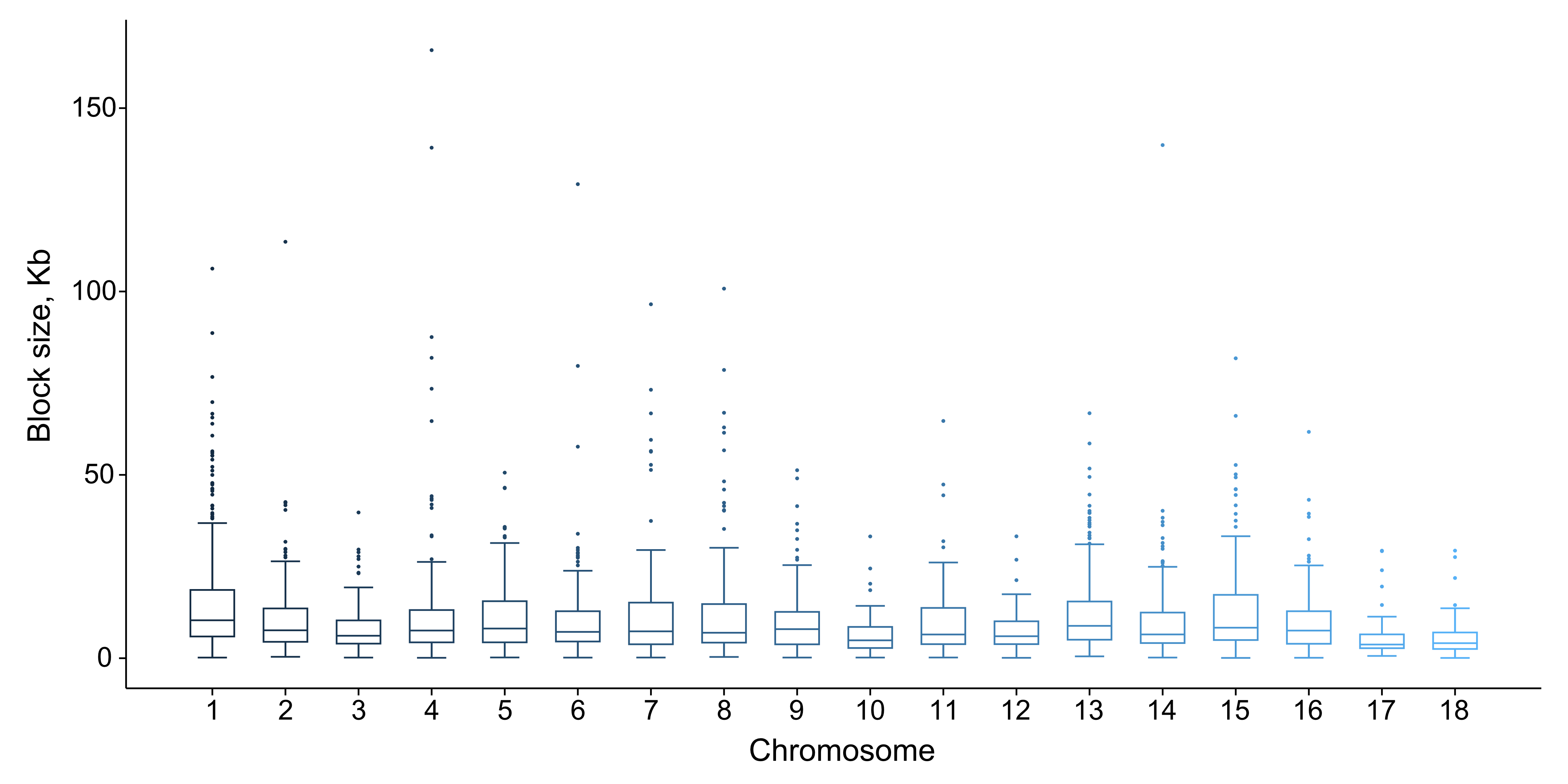
**

**Fig. S2.** The block size distribution for each autosome in our data.

**
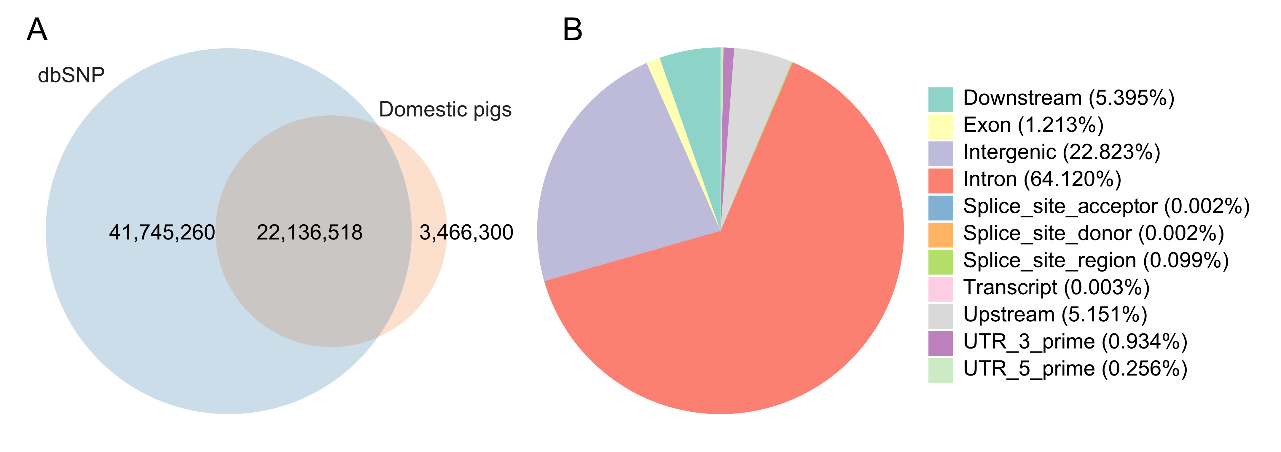
**

**Fig. S3.** Genetic variants of 82 genomes from six local Chinese pig breeds (Ding’an pigs; Hetao pigs; Min pigs; Tibetan pigs; Tunchang pigs; Wuzhishan pigs). **A** Venn diagrams for novel variants detected in Chinese domestic pigs. **B** Annotation of 25,602,818 SNPs retrieved from Chinese domestic pigs.

**
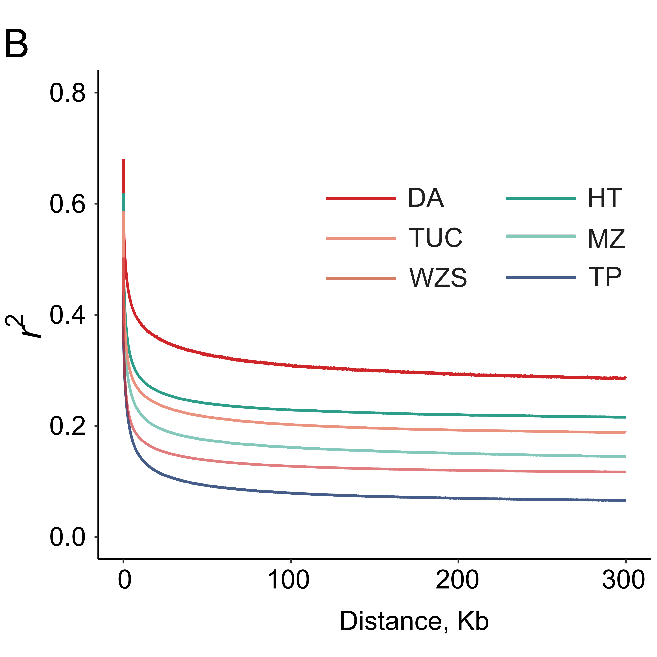
**

**Fig. S4.** Decay of Linkage disequilibrium (LD) for six breeds, with one line per breed. (DA, Ding’an pigs; HT, Hetao pigs; MZ, Min pigs; TP, Tibetan pigs; TUC, Tunchang pigs; WZS, Wuzhishan pigs).

**
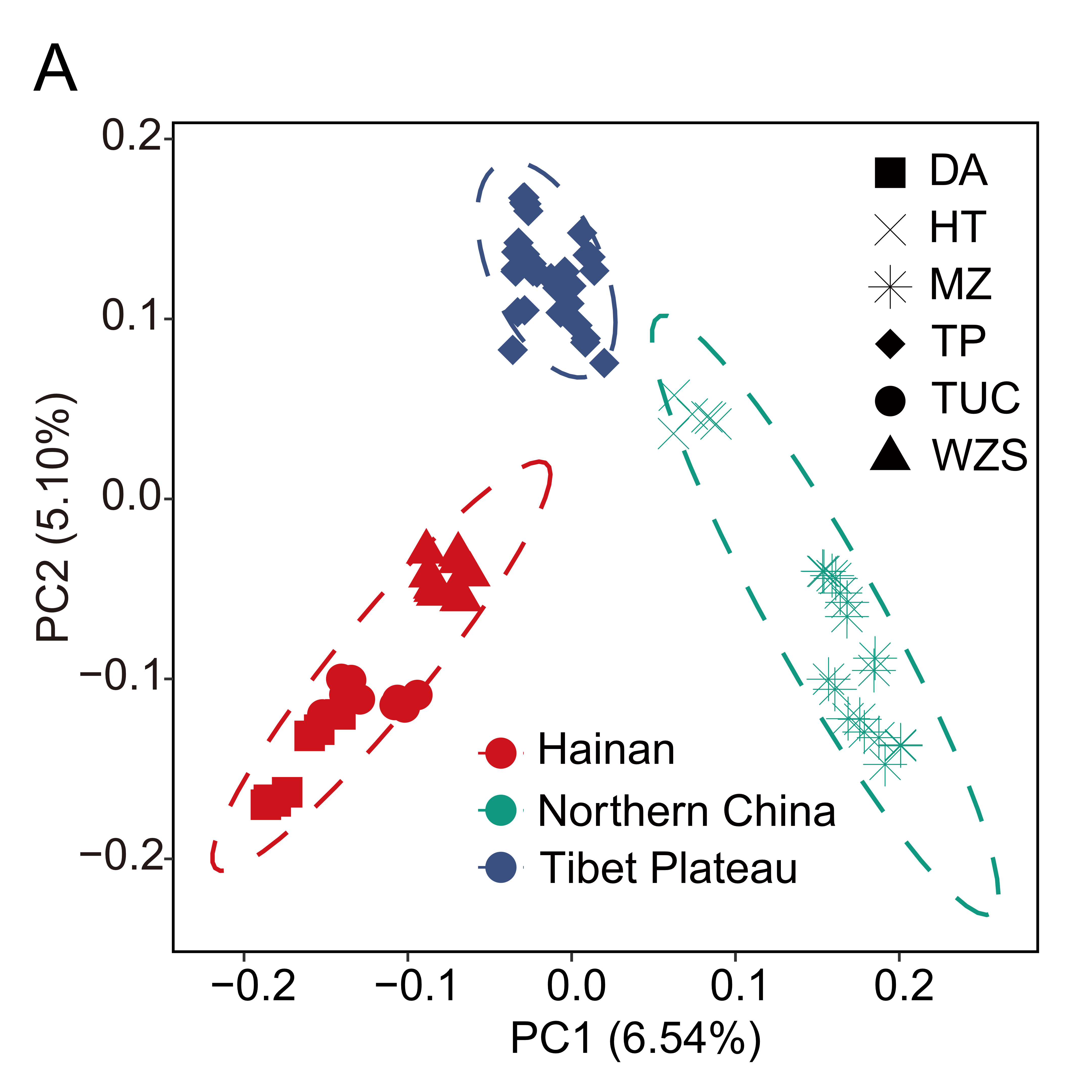
**

**Fig. S5.** A principal component plot of the 82 individuals based on SNP information. The color represents the location of the pig breeds. DA, Ding’an pigs; HT, Hetao pigs; MZ, Min pigs; TP, Tibetan pigs; TUC, Tunchang pigs; WZS, Wuzhishan pigs.

**
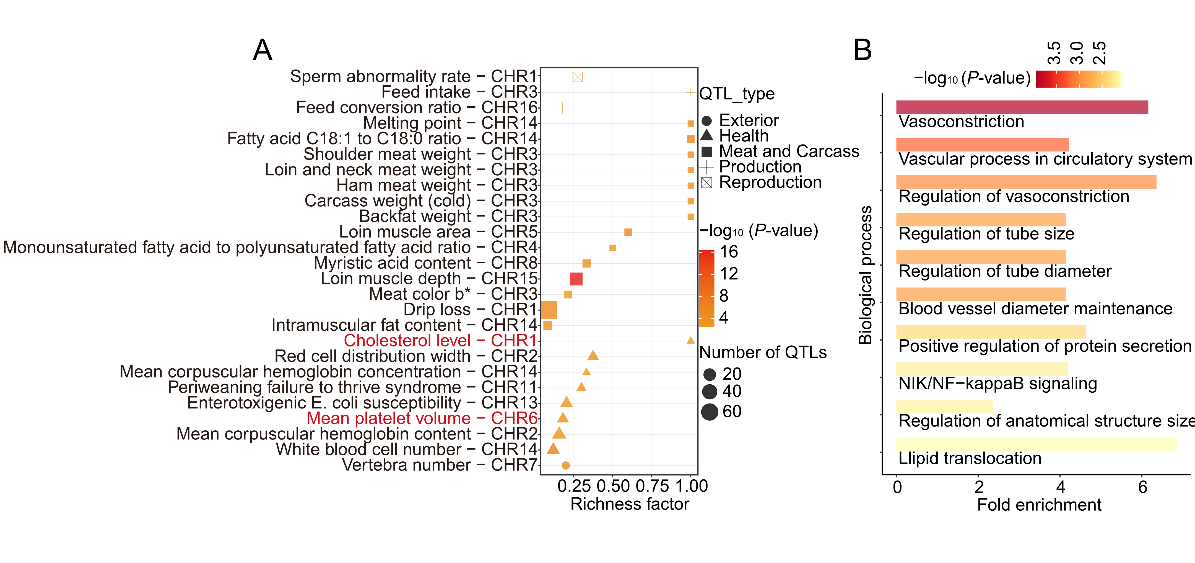
**

**Fig. S6.** Annotation of the regions and genes under tropical adaptation-specific selection based on the Animal QTLdb and the Gene Ontology Resource, respectively. **A** Significantly enriched QTL terms for tropical-specific selection. The richness factor was obtained by calculating the ratio of the number of QTLs annotated in the candidate regions and the total number of each QTL. **B** Significantly enriched GO terms (Biological process, top 10) for tropical-specific selection.

**
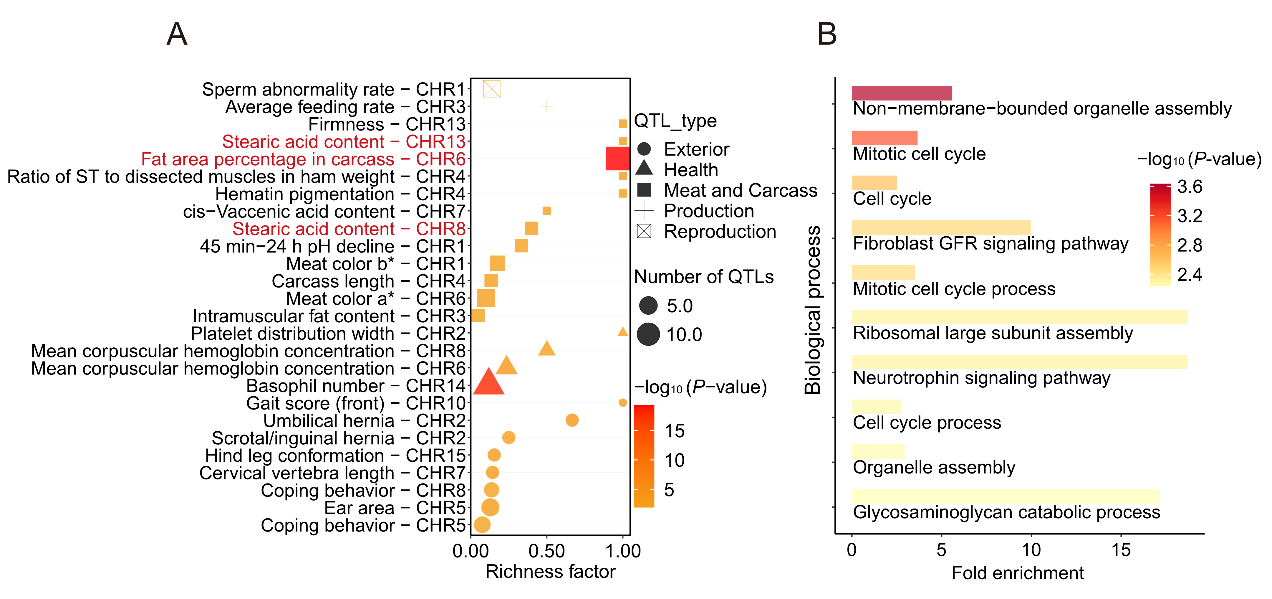
**

**Fig. S7.** Annotation of the regions and genes under frigid adaptation-specific selection based on the pig QTLdb and the Gene Ontology Resource, respectively. **A** Significantly enriched QTL terms for frigid-specific selection. The richness factor was obtained by calculating the ratio of the number of QTLs annotated in the candidate regions and the total number of each QTL. **B** Significantly enriched GO terms (Biological process, top 10) for frigid-specific selection.

**
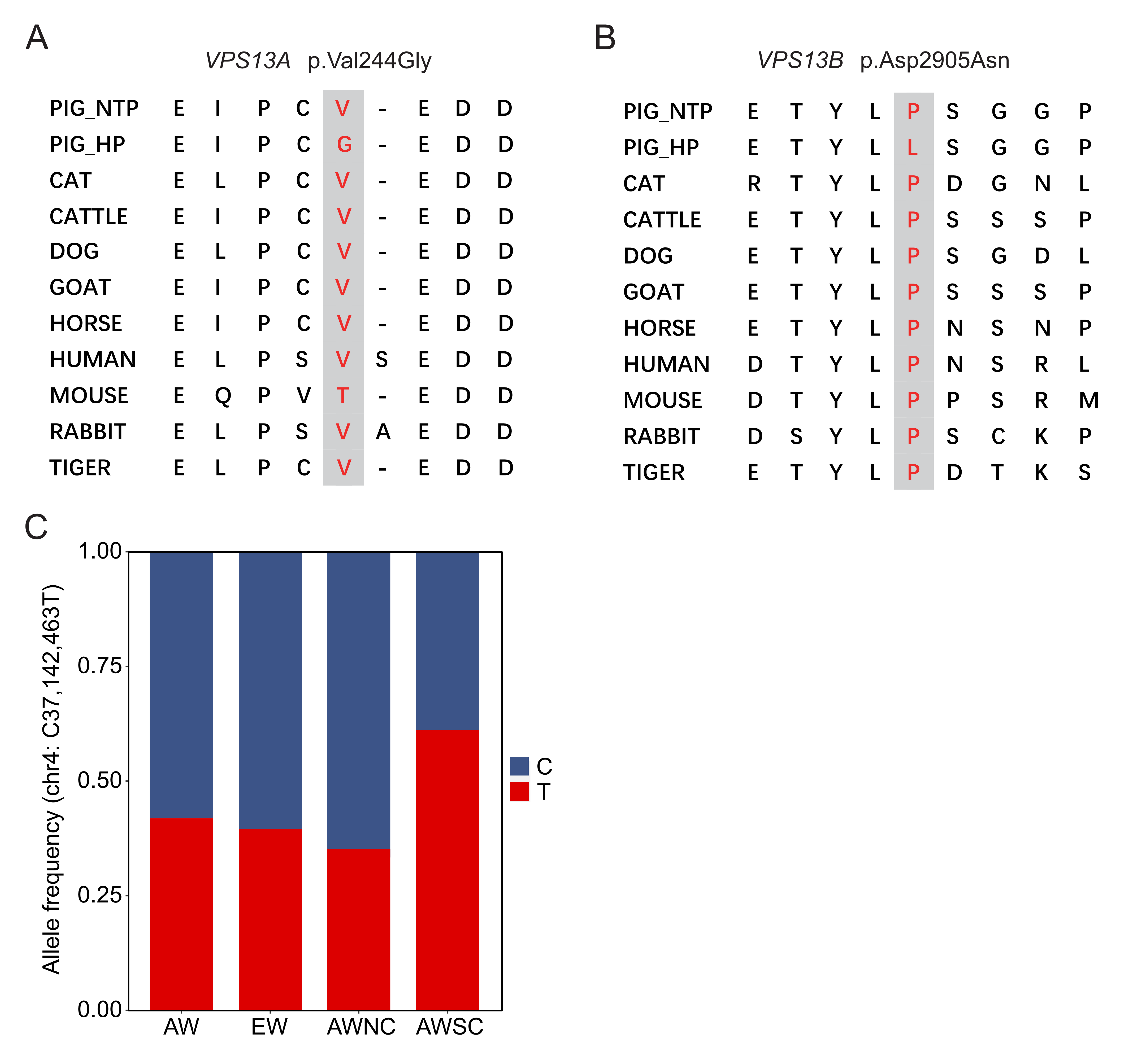
**

**Fig. S8.** Multispecies regional alignment of the *VPS13A* and *VPS13B* protein sequences around candidate variants for adaptation to tropical environments. **A** p.Val244Gly in *VPS13A* gene. **B** p.Asp2905Asn in *VPS13B* gene. **C** Allele frequency of the candidate variant in *VPS13B* among different breeds. AW, Asian wild pigs; EW, European wild boars; AWNC, Northern Chinese wild boars; AWSC, Southern Chinese wild boars.

**
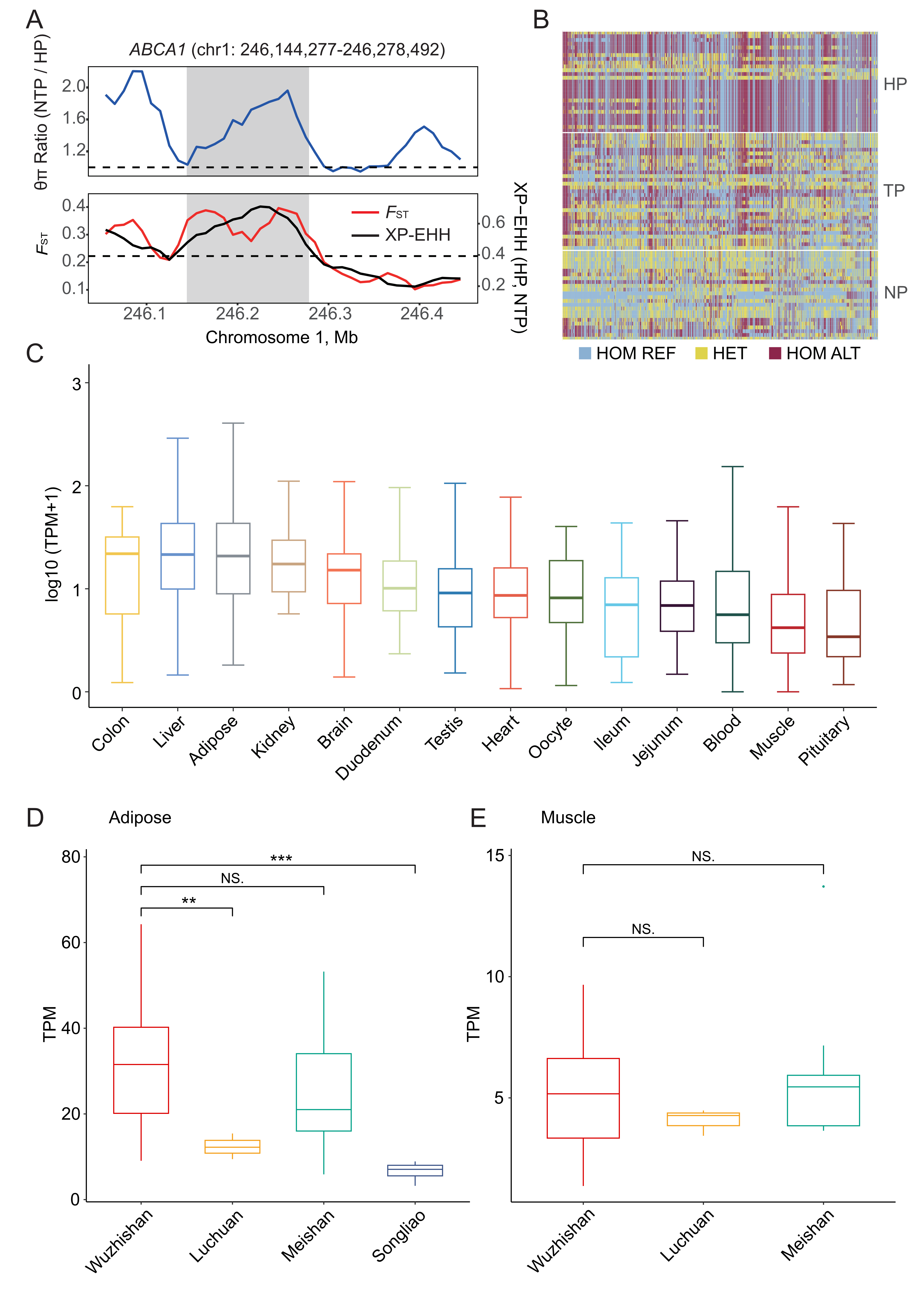
**

**Fig. S9.** Distinct genomic landscape and expression levels of *ABCA1* gene. **A** θπ ratios (50-kb windows, 10-kb steps), *F*_ST_ values, and XP-EHH values around the *ABCA1* gene locus. The blue line represents θπ ratios. The red and black lines represent *F*_ST_ and XP-EHH values, respectively. **B** Haplotype pattern in the genomic region of *ABCA1* among HP, TP, and NP. **C** The *ABCA1* gene shows predominant expression in liver tissues. **D** and **E** Expression of *ABCA1* gene in adipose and muscle tissues of different pig breeds. From left to right, the average annual temperature of pig breeding areas is decreasing. The significance of the genotype difference is tested. ** *P* < 0.05 (t-test), *** *P* < 0.001 (t-test).

**
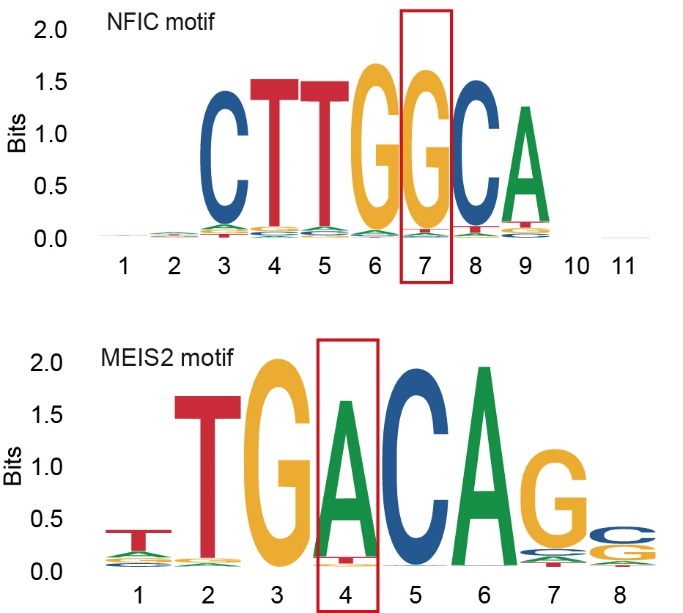
**

**Fig. S10.** Sequence logos of *NFIA* and *MEIS2*. Site 7 (G allele) of *NFIA* and site 4 (A allele) of *MEIS2* are the binding sites of chr1: 246,175,129.
